# Complete mitochondrial genome and the phylogenetic position of the Borneo leg skate *Sinobatis borneensis* (Rajiformes: Anacanthobatidae)

**DOI:** 10.1080/23802359.2016.1180560

**Published:** 2016-07-08

**Authors:** Ranran Si, Wenyong Ding, Weiming Ai, Xiao Chen

**Affiliations:** a Department of Marine Science, Wenzhou Medical University, Wenzhou, Zhejiang, China;; b Zhejiang Mariculture Research Institute, Wenzhou, Zhejiang, China

**Keywords:** Anacanthobatidae, rajiformes, *Sinobatis borneensis*

## Abstract

In this study, the complete mitochondrial genome of the Borneo leg skate *Sinobatis borneensis* (Rajiformes, Anacanthobatidae) was determined. It had circular molecules (16,701 bp), consisting of 37 genes with a typical gene order in vertebrate mitogenome. In the whole mitogenome, there were 28 bp short intergenic and 31 bp overlaps, respectively, located in 12 and 7 gene junctions. The nucleotide composition was 31.1% A, 26.0% C, 13.9% G and 29.1% T. Two start codons (GTG and ATG) and two stop codons (TAG, TAA/T) were used in the protein-coding genes. The 22 tRNA genes ranged from 66 bp (tRNA-*Cys*) to 75 bp (tRNA-*Leu1* and tRNA-*Lys*). The phylogenetic result showed that *S. borneensis* was clustered with the *Atlantoraja castelnaui* and *Pavoraja nitida*.

The Borneo leg skate *Sinobatis borneensis* was distributed in the Western Pacific, from South China Sea to Taiwan (Last & Séret 2008). It had typical morphometrics with thin disc, broad snout, slender tail, small eye and long rostral filament (McEachran & Dunn 1998). In this study, we determined the complete mitochondrial genome of *S. borneensis* for the first time and analyzed the phylogenetic relationship in Rajiformes.

One specimen of *S. borneensis* was captured in the South China Sea, and preserved in the Museum of Marine Biology in Wenzhou Medical University with voucher NH20110605. The experimental protocol and data analysis methods followed Chen et al. (2015). Including *S. borneensis*, 12 species of Rajiformes, with the complete mitogenomes available in the Genbank, were selected to construct the phylogenetic tree using Bayesian method. The outgroup were *Dasyatis akajei* and *D. bennetti* (Myliobatoformes).

The complete mitochondrial DNA of *S. borneensis* was 16,701 bp, consisting of 13 protein-coding genes, 22 tRNA genes, 2 rRNA genes and 1 non-coding control region (Genbank accession No. KX014715). Except for *ND*6 gene and eight tRNA genes (tRNA-*Gln*, *Ala*, *Asn*, *Cys*, *Tyr*, *Ser1, Glu,* and *Pro*), all other genes were encoded on the H-strand. In the whole mitogenome, there were 28 bp short intergenic and 31 bp overlaps, respectively, located in 12 and 7 gene junctions. The nucleotide composition was 31.1% A, 26.0% C, 13.9% G, and 29.1% T, with a relatively lower level of G and a slight A-T bias of 60.2%. Two start codons (GTG and ATG) and two stop codons (TAG, TAA/T) were used in the protein-coding genes, and most of them shared common initial codon ATG and terminal codon TAA. The *COI* gene used non-standard initial codon GTG as well as most other vertebrates (Slack et al. 2003). The *COII* and *ND4* genes were terminated with a single T, which could be extended to complete TAA through polyadenylation after transcriptions (Ojala et al. 1981). The 22 tRNA genes were ranged from 66 bp (tRNA-*Cys*) to 75 bp (tRNA-*Leu1* and tRNA-*Lys*). Both 12S and 16S rRNA genes were located between the tRNA-*Phe* and tRNA-*Leu1* genes, separated by the tRNA-*Val* genes. A 37 bp inserted sequence was identified as the origin of light-strand replication (OL) between tRNA-*Asn* and tRNA-*Cys* genes with a stem-loop structure. The control region was 1060 bp, presenting a high A + T content (64.8%).

Most nodes were strongly supported in the Bayesian tree (Figure 1). Within the Rajiformes, 12 available species belonged to three families (Rhinobatidae, Rajidae and Arhynchobatidae). The topology showed that three families and all genus were monophyletic. The main division was between Rhinobatidae and (Arhynchobatidae + Rajidae) clade, which consistented to the morphological result (McEachran & Dunn 1998). Within Arhynchobatidae, *S. borneensis* was sister to the *Atlantoraja* +* Pavoraja* clade.

**Figure 1.**
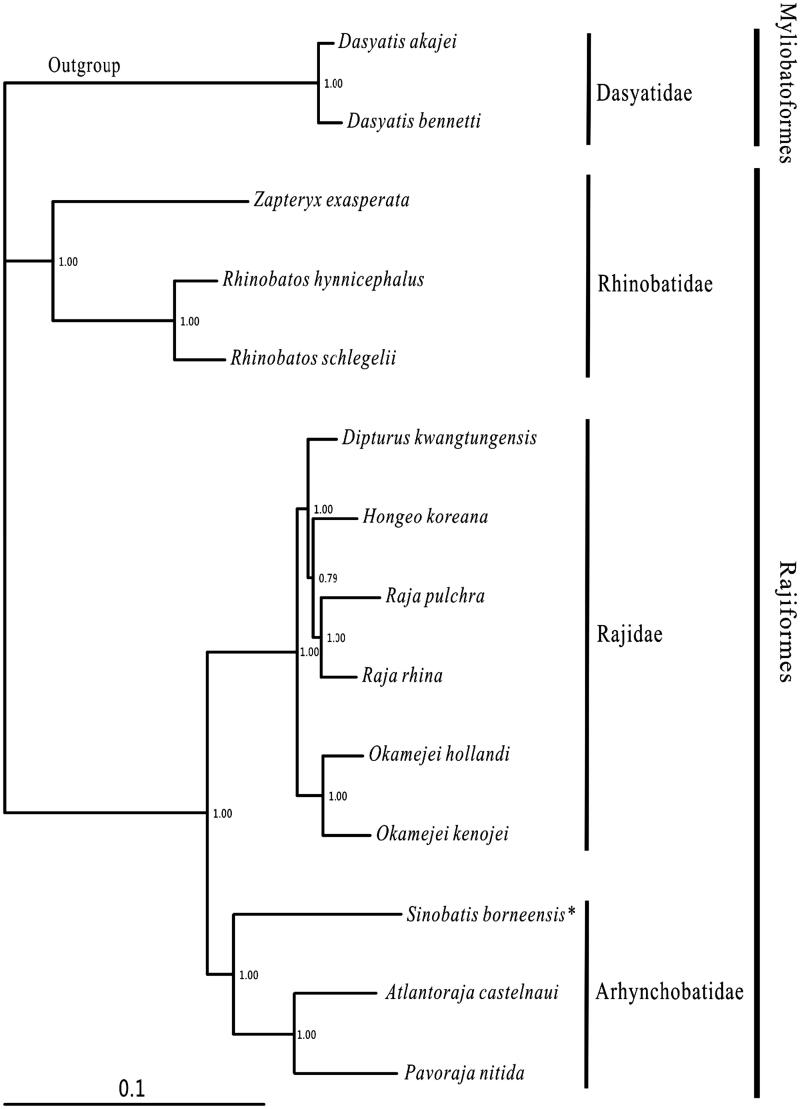
Phylogenetic position of *Sinobatis borneensis*. *Dasyatis akajei* (NC_021132.1) and *D. bennetti* (KC633222.1) were selected as the outgroup. The twelve species of Rajiformes were *Zapteryx exasperata* (NC_024937.1), *Rhinobatos hynnicephalus* (NC_022841.1), *R. schlegelii* (NC_023951.1), *Dipturus kwangtungensis* (NC_023505.2), *Hongeo koreana* (NC_021963.1), *Raja pulchra* (NC_025498.1), *Raja rhina* (KC914434.1), *Okamejei hollandi* (KP756687.1), *O. kenojei* (NC_007173.1), *Sinobatis borneensis* (KX014715), *Atlantoraja castelnaui* (NC_025942.1) and *Pavoraja nitida* (NC_024599.1).
